# A narrative review of AI-driven stroke rehabilitation systems through the lens of human motor learning

**DOI:** 10.3389/fneur.2026.1800204

**Published:** 2026-04-24

**Authors:** Zakaria Taghi, Deborah J. Serrien, Lucas Fonseca, Nikhil Deshpande

**Affiliations:** 1 School of Computer Science, University of Nottingham, Nottingham, United Kingdom; 2 School of Psychology, University of Nottingham, Nottingham, United Kingdom

**Keywords:** AI, assistive technology, human motor learning, machine learning, neurorehabilitation, robotics, VR/AR

## Abstract

**Introduction:**

Stroke represents a leading global cause of disability, often causing motor impairments that diminish quality of life. Neurorehabilitation that leverages human motor learning (HML) theories is crucial for post-stroke recovery. Therapists guide repetitive practice that supports re-learning, and they adjust assistance to individual needs and progress. Robot-assisted rehabilitation has advanced this approach, and recent work shows AI-driven systems can improve adaptability to patient behavior beyond earlier technologies. However, notably few systems aim to explicitly replicate therapist assistance from the perspective that physical assistance is a motor skill in itself.

**Methodology:**

This narrative review examines advances in AI-driven stroke rehabilitation, analyzing how systems facilitate HML within patients and how their models approximate HML mechanisms. By breaking down the four core HML processes to their essentials, using Marr's tri-level hypothesis, we compare machine learning models used within rehabilitation systems to the HML processes.

**Results:**

Many of the reviewed systems appear to primarily facilitate use-dependent and sensory-prediction error-based learning, with limited facilitation of reinforcement learning or strategy-based learning. Explicit modeling of therapist HML within control frameworks appears relatively rare. Implicitly, many of the reviewed AI systems functionally represent one or two HML processes.

**Conclusion:**

Current research often considers HML primarily in patients, whereas therapists' own HML likely underpins the robustness and adaptability of clinical assistance. Interpreting the reviewed rehabilitation systems through this lens highlights opportunities for therapist-inspired multi-process controllers, improved benchmarking with clinical scales, longitudinal retention studies, and AI-driven closed-loop neuromodulation to enhance personalization, adaptability, and outcomes, and to support clinical translation into routine practice.

## 1 Introduction

Stroke is a leading cause of mortality and disability across the world, being in the top 10 leading causes of disability adjusted life years (DALYs) (1). There are two main types of stroke: ischemic stroke, caused by a blockage of a cerebral artery, and hemorrhagic stroke, resulting from the rupture of a blood vessel. These cause brain damage that can range from mild to catastrophic, resulting in impairments in movement, perception, speech, and executive function. Further complications, such as apathy and emotional dysfunction, can arise as well that require treatments of their own (2, 3). The effects of these impairments can be reduced with the aid of assistance and rehabilitation (4). Therefore, the right rehabilitation is crucial in recovery from stroke. The post-stroke state of a patient can be separated into five phases: hyper-acute (24h after), acute (7 days after), early sub-acute (1–3 months after), late sub-acute (4–6 months after), and chronic (6+ months after) (5). Effective rehabilitation during the early phases has been found to be the most effective in long-term stroke recovery (6).

Different approaches to rehabilitation exist, and the type of rehabilitation method used varies depending on a variety of factors, some of which include therapist experience, patient ability, equipment availability, and severity of stroke (7). However, the majority of methods are grounded in theories of HML and the best ways to facilitate and optimize this process (8). Built upon traditional rehabilitation principles, rehabilitation technology has been developed and has proven capable of being used to improve motor performance within patients (9). Furthering the development of rehabilitation technology is research into the inclusion of AI and machine learning (ML) models to drive these systems, prospectively allowing for improved specificity to patients as well as greater context-awareness.

Despite recent advances, evidence from large trials and meta-analyses indicates that technology-assisted stroke rehabilitation typically yields outcomes comparable to dose-matched conventional therapy, and consistent superiority has not been demonstrated across modalities and clinical scales (10, 11).

Therapists adapt assistance continuously in response to patient performance and behavior, enabling highly personalized rehabilitation. We hypothesize that the robustness of therapist assistance arises from *therapist HML*—the learning processes therapists themselves employ to refine strategies and adjust assistance based on their formal training, experience over the years, and across repeated interactions with patients. Despite its potential importance, this aspect of rehabilitation remains under-represented in the design of current AI-driven rehabilitation systems (12).

Therapist-in-the-loop controllers represent one step toward incorporating learnt therapist expertise into rehabilitation technologies. However, these approaches still require therapists to remain actively involved in the control loop, limiting the time efficiency and scalability that fully autonomous rehabilitation systems aim to achieve (13). Building upon this, assist-as-needed (AAN) approaches attempt to reproduce therapist-like adjustments autonomously by modulating assistance according to measures such as task error or performance success (14). Whether implemented through pre-defined heuristics or through learned models, these systems are designed with reference to what therapists do, the adjustments they make, rather than the processes through which they learn to do it. Therapist adjustment and therapist learning are distinct: the former describes observable behavior, the latter describes the acquisition and refinement of the expertise that produces that behavior. This distinction has not been addressed in the rehabilitation robotics literature, where adaptive controllers are evaluated on the basis of their outputs rather than the correspondence between their underlying mechanisms and those of human clinical learning.

This review examines AI-driven rehabilitation systems from a different perspective: rather than evaluating how closely system outputs resemble therapist behavior, it considers whether the underlying computational mechanisms correspond to the learning processes through which therapists develop clinical expertise. This question has direct relevance to two challenges that the rehabilitation robotics literature identifies as unresolved: (i) the personalization of assistance to individual patients, and (ii) the development of systems capable of adapting reliably across the heterogeneous patient populations encountered in clinical practice (14). Therapist expertise has been characterized as involving multiple interacting processes operating across different timescales, including the consolidation of skills through extended clinical practice and within-session adjustments in response to patient performance (7), processes that map onto both of these challenges. Whether AI-driven systems that more closely correspond to these mechanisms are better positioned to address the challenges, remains an open question, but identifying where such correspondences exist and where they do not provides a principled framework for evaluating current limitations and directing future development in ways that purely behavioral comparisons cannot.

This review aims to cover the body of recent research resulting in AI-driven stroke rehabilitation systems and analyze them for how they facilitate HML, as well as whether they model processes of HML themselves, to aid in the rehabilitation process.

## 2 Background

### 2.1 Human motor learning

HML refers to the processes involved in the acquisition and adaptation of motor skills through practice and experience. These processes lead to relatively permanent changes in skilled actions, such as improvements in speed, accuracy, and coordination. HML is shaped by sensory feedback, cognitive processes, neural adaptation, and memory, all of which contribute to neural plasticity (15). Some of these adaptations occur implicitly, and some occur explicitly through conscious processing and decision-making used to learn a motor skill or complete the current task (16). HML theories can be sectioned into two broad groups: (i) frameworks of HML and (ii) processes of HML. HML frameworks describe the phases a person goes through as they acquire motor skills, whereas HML processes refer to consistent dynamics that occur throughout learning. The neuro-dynamical basis underlying these theories is outside the scope of this review.

### 2.2 Frameworks of HML

The frameworks of HML generally align with one of three paradigms: the cognitive approach, the ecological approach, and the dynamical systems approach. The cognitive approach focuses on internal processing and the handling of new information (17). The ecological approach centeres on the interaction between the individual and the environment, with environmental context influencing how motor skills are learned (18). Dynamical systems theory (DST) views motor learning as a self-organizing process shaped by constraints from the individual, environment, and task (19).

The Fitts and Posner 3-stage model aligns with the cognitive approach and describes learning as progressing from high cognitive engagement to automaticity (20).

1. *Cognitive stage:* the learner relies on conscious problem-solving to identify task goals and determine how to act within the environmental conditions.
2. *Associative stage:* feedback is used to refine movement and improve consistency while adapting actions to the environment.
3. *Autonomous stage:* the motor skill is executed fluidly with minimal cognitive load, allowing efficient adaptation to environmental variations.

The Bernstein 3-stage model aligns with the dynamical systems approach and focuses on the degrees-of-freedom (DoFs) of the body used during learning (21).

1. *Freezing:* the learner simplifies control by restricting certain DoFs to stabilize movement.
2. *Exploring:* previously restricted joints are gradually released as new coordination patterns are explored.
3. *Exploiting:* the learner utilizes the mechanical properties of the body to maximize performance and efficiency.

The Gentile 2-stage model aligns with the ecological approach and emphasizes environmental variability (22).

1. *Initial stage:* the learner identifies environmental features that influence the movement.
2. *Later stage:* for closed skills the goal is fixation, whereas for open skills the goal is diversification, adapting movement to changing environments.

Together, these frameworks represent the cognitive, ecological, and dynamical systems perspectives of motor learning (see Table 1).

**Table 1:** The main HML frameworks, their stages, and how learner state, task goals, and environmental context interact.

Framework	Stages	Learner's cognitive state and task goals	Role of environmental context
Fitts and Posner (20)	Cognitive, associative, autonomous	Moves from conscious problem-solving to automated execution; goal shifts from figuring out “what to do” to optimizing “how to do it.”	Environment initially requires explicit analysis, later adapted to automatically.
Bernstein (21)	Freezing, exploring, exploiting	Early simplification of control; goal shifts to exploiting body mechanics for efficiency.	Environmental constraints guide exploration and selection of stable coordination patterns.
Gentile (22)	Initial, later—fixation or diversification	Initial focus on distinguishing relevant vs. irrelevant environmental cues; later goal is stability or adaptability depending on environmental stability.	Central to defining regulatory cues and shaping adaptation strategies.

Advances in theory and technology have also led to newer frameworks of HML. One such framework is Optimal Feedback Control (OFC), which proposes that the motor system generates actions by minimizing expected task-specific costs such as effort or error while using sensory feedback selectively to correct movement (23). This computational perspective begins to bridge theoretical models of human motor behavior with computational approaches used in robotics and control.

### 2.3 Processes of HML

HML processes drive motor skill acquisition and adaptation and occur throughout the stages described above (see Table 2).

**Table 2:** The core HML processes, their drivers and brief descriptions.

Process	Driver	Description	Main neural correlate
Reinforcement learning	Reward prediction error	Learning based on the expected reward of an action (calculated within dopaminergic circuits) and the actual reward	Basal Ganglia (98)
Sensory prediction error (SPE)-based learning	Sensory error	Learning based on the difference between the expected sensory input and the actual sensory input	Cerebellum (99–101)
Strategy-based learning	Cognitive control	Learning based on the outcomes of formulated strategies and their success in completing a task	Prefrontal Cortex (102, 103)
Use-dependent learning	Movement repetition	Learning based on the process of repeatedly carrying out a movement pattern	Primary motor cortex (15, 33)

One core process is *reinforcement learning* (RL), which enables learning from success and failure when performing a motor task (24). Behavior is modified based on the difference between expected and actual task success. Neurophysiological evidence links reinforcement learning to dopamine-mediated reward signals that strengthen synaptic connections associated with successful actions (25, 26).

Another key process is *sensory prediction error* (SPE) learning (27). SPE occurs when the predicted sensory consequences of a movement differ from the sensory feedback received, driving adaptation of motor commands. This process plays an important role in adapting to perturbations and recalibrating internal models (27, 28). Evidence also suggests that SPE-based learning can contribute to internal model consolidation and inter-limb transfer of learning (29, 30).

*Strategy-based learning* represents a more explicit learning process. Unlike reinforcement learning and SPE, which are largely implicit, strategy-based learning involves consciously selecting and adjusting actions based on goal-performance errors (31). External feedback such as auditory or visual cues can facilitate strategy adjustment and explicit reinforcement processes (24). Neurophysiological studies associate this process with changes in post-movement β oscillations in sensorimotor cortex linked to error evaluation and internal model updating (32).

The final core process is *use-dependent learning* (33). This form of learning is driven primarily by repetition rather than feedback. Through repeated execution of a movement, motor behavior becomes biased toward previously performed actions, resulting in gradual but persistent changes in movement patterns.

Other mechanisms can modulate these processes. *Observational learning* and *motor imagery* activate motor networks without overt execution and can facilitate motor learning through action observation and mental rehearsal (34, 35). Context inference, attention, and cognitive load can also influence how reinforcement, SPE, and strategy-based processes contribute to motor learning (36–38).

### 2.4 Rehabilitation technology and HML

Research into HML provides a strong theoretical foundation for rehabilitation methods. Some of these, e.g., proprioceptive neuromuscular facilitation and mirror therapy, leverage processes such as use-dependent learning and motor imagery to support rehabilitation (39, 40). Whereas, methods such as the neurodevelopmental approach apply techniques that stimulate processes including SPE-based learning (41). Following on from more traditional approaches, there is a vast body of research exploring how technology can be used to aid in the rehabilitation process. The primary rehabilitation technologies that this review will cover are assistive robotics, virtual reality and augmented reality (VR & AR), neurostimulation and vision-based systems. Brain computer interfaces (BCIs) were also initially considered as a stand-alone category. However, BCIs do not inherently have a physical embodiment, and thus, BCI systems cannot directly facilitate motor learning. Nonetheless, it may be used as a sensor modality for driving the systems mentioned (42).

Robot-assisted therapy, encompassing both end-effector-based devices and robotic exoskeletons, provides standardized, intensive, and repetitive movement training to patients following stroke (43). By facilitating high-intensity and precise motor repetitions, these systems leverage neuroplasticity to promote motor recovery to a similar level of traditional therapy, closely aligning with the principles of use-dependent learning (44). Many existing robotic systems implement fixed assistance strategies, rarely adapting dynamically in response to clinicians' performance changes or variability during therapy sessions. This rigidity can result in suboptimal personalization and may limit the full potential of recovery (45).

Virtual rehabilitation technologies, including virtual reality (VR) & augmented reality (AR), create immersive, interactive environments designed to enhance patient engagement and motivation (46). These technologies can promote motor recovery by providing multisensory feedback and realistic task-specific scenarios that facilitate strategy-based and SPE-based learning (47). They have proven to be especially effective in improving motor function in stroke populations with moderate to severe upper limb paresis (48). However, despite their advantages, VR and AR systems often employ generic scenarios with limited adaptive capabilities, rarely adjusting complexity or assistance in real-time based on individualized patient responses. The system itself does not typically contribute to the treatment plan and thus, this is still directed by the clinicians judgment (49). Consequently, a limitation of them is their lack of ability to produce rehabilitation protocols without clinician input (50).

Neuromodulation techniques, including Functional Electrical Stimulation, Transcranial Direct Current Stimulation, and Transcranial Magnetic Stimulation, aim to modulate neural excitability and enhance cortical plasticity, thereby promoting motor recovery post-stroke (51, 52). These approaches theoretically complement motor training by priming neural pathways for enhanced plasticity. However, neurostimulation protocols vary between studies, with a smaller subset using adaptive closed-loop systems as opposed to open-loop systems (53, 54).

## 3 Machine learning within this review

In this review, we focus specifically on AI-driven rehabilitation systems that actively influence therapy delivery, for example by modifying robotic assistance, adjusting task difficulty, or altering feedback during task execution (12). Systems that apply machine learning solely for assessment, diagnosis, prognosis, or *post-hoc* analysis of patient performance are excluded, as they do not directly influence therapeutic interaction and therefore do not participate in learning during rehabilitation.

Machine learning broadly refers to computational approaches that learn relationships between input data and outputs through experience (55). In rehabilitation systems, the inputs to these models typically consist of sensorimotor signals such as joint kinematics, interaction forces, EMG activity, or visual tracking data (56). The outputs produced by these models may include movement classifications, estimates of patient intention, or adaptive changes to assistance levels and task difficulty during task execution (14). The models used across reviewed systems span several broad paradigms, ranging from supervised learning approaches that learn from labeled examples, to probabilistic models that represent uncertainty explicitly, to reinforcement learning methods that derive policies through interaction.

Artificial neural networks (ANNs) are commonly employed to learn non-linear relationships between sensorimotor inputs and system outputs through iterative weight optimization (57). Within this family, convolutional neural networks (CNNs) are often used to extract features from spatial or time-series data, while recurrent architectures such as long short-term memory networks (LSTMs) and recurrent neural networks (RNNs) model temporal dependencies in sequential signals. More recently, transformer-based models have also been explored due to their ability to capture long-range dependencies using attention mechanisms (57). Some systems extend these architectures into hybrid structures: neuro-fuzzy approaches, for instance, integrate neural network learning with fuzzy logic rules, allowing rule-based systems to be adapted automatically from data rather than defined manually (58), offering a balance between adaptability and interpretability.

A number of classical machine learning approaches frame the problem as classification or regression within a structured feature space. Methods such as support vector machines (SVMs) and linear discriminant analysis (LDA) identify decision boundaries that separate classes in feature space, either through maximally separating hyperplanes or through projections that maximize class separability (59). Instance-based approaches such as k-nearest neighbors (K-NN) instead classify new samples according to the labels of the most similar observations in the training dataset, using distance metrics within the feature space. Tree-based ensemble methods adopt a different strategy by partitioning the input space through feature thresholds: random forests aggregate predictions across many decision trees to improve generalization, while gradient boosting methods, including Extreme Gradient Boosting (XGBoost), iteratively refine predictions by fitting new trees to the residual errors of previous models (60).

Other approaches rely on probabilistic models that explicitly represent uncertainty in the learned relationships. Gaussian processes (GPs) and Gaussian mixture models (GMMs) learn probability distributions over data rather than deterministic mappings, making them particularly suited to trajectory modeling and contexts where uncertainty estimation is important (61, 62). These properties make them a natural fit for rehabilitation applications involving smooth, continuous movement.

RL represents a distinct paradigm in which an agent learns behavioral policies through trial-and-error interaction with an environment, updating its actions in order to maximize cumulative reward rather than learning from labeled examples (63). In rehabilitation robotics, RL methods are often used to learn control policies that adjust assistance levels or movement trajectories based on patient performance. Deep reinforcement learning extends this framework by using neural networks to approximate value functions or policies in high-dimensional state spaces, with algorithms such as proximal policy optimization (PPO), soft actor-critic (SAC) and Relative Entropy Policy Search (REPS) being used (63).

A defining characteristic of AI-driven rehabilitation systems is that they operate within a closed-loop interaction with the patient: model outputs directly influence assistance, feedback, or task parameters, which in turn alter patient behavior and generate new sensory inputs (14). Within clinical practice, therapists perform an analogous process, applying methods previously learnt through training while continually adjusting assistance and feedback in response to patient performance (7).

## 4 Methodology

The conceptual outlines of the HML processes within the background do not permit objective categorization of machine learning models. A better comparison between complex HML processes and machine learning models can start to be made by distilling the HML processes down to their fundamentals (Also shown in Table 3):

**Table 3:** HML process categorization.

Process	Learning signal	Updated component	Observable indicator
Use-dependent learning	Repetition and recency	Action bias	Gradual shift toward recently/frequently used actions
Reinforcement learning	Reward/return	Policy/value function	Gradual bias toward high-reward actions
Strategy-based learning	Goal performance error	Explicit action-selection rule, offset or mode switch	Rapid correction with minimal carry over upon task-switching
SPE-based learning	Prediction error	Internal forward/inverse model	Gradual recalibration, after-effects when task changes

Use-dependent learning, functionally, is driven by repetition and recency, largely independent of evaluative feedback. It is best characterized as frequency-biased reuse of prior actions or mappings and thus models updated purely through data frequency would align most strongly.

SPE-based learning corresponds to updating internal forward/inverse models using sensory prediction error; the defining feature is the prediction-error signal, not the specific network architecture. Typically feed-forward models such as traditional NNs, CNNs, and RNNs align most strongly with this, providing as good examples of the type of model that would be categorized as an “SPE-based learning”-like model.

Strategy-based learning functions through explicit action selection and is driven through goal-performance error. Where SPE-based learning is implicit and updates the internal model of the world, strategy-based learning is explicit and instead results in explicit action-selection rules, for example, adding an aiming offset or switching to a pre-defined command, so as to reduce goal error without changing the internal mapping; the corrections are quick and deliberate, with little carry-over once the rule is removed. This functions most similarly to rule-based machine learning models such as decision-trees and neuro-fuzzy models, due to explicit rule-based nature of these models.

Reinforcement learning has the clearest link to through reinforcement learning models, a prominent paradigm within machine learning. Centered upon the presence of a reward signal, actions with the highest predicted reward to be selected, functioning with very strong similarity to reinforcement learning within HML.

However, a key consideration in comparing machine learning models to HML processes is that HML processes are defined by their function over their form. Therefore, a machine learning model such as an ANN can be functionally represent SPE-based learning when used for intention prediction, while also being functionally representing strategy-based learning when used as part of a Neuro-fuzzy system. Taking this point into consideration, we have utilized Marr's tri-level hypothesis in order to more objectively categorize machine learning models as representing individual HML processes. Marr's hypothesis proposes that any information-processing system can be understood through three complementary levels of analysis (64). The computational level defines what the system is doing and why, the goal of the computation and the problem it seeks to solve. The algorithmic level describes how this computation is carried out, specifying the representations and processes that transform inputs into outputs. The implementation level focuses on how these representations and processes are physically realized in a particular substrate, such as biological neural tissue or artificial hardware.

By applying the definitions of the computational and algorithmic levels, a rubric can be established for each HML process. This enables a clearer and more systematic classification of machine learning models based on their goal, operationalised as the primary learning signal that drives adaptation, and their function, operationalised as the component within the model that is updated during learning. The third level of Marr's framework is not used within this categorization framework as most computational models operate on fundamentally different principles from organic neural networks thus comparison of architecture and implementation would not be appropriate. This exception does not extend to spiking neural networks, which more closely emulate biological neural mechanisms, however, no primary research reviewed employed spiking models.

Papers for this review were collected from the Scopus, PubMed, IEEE Xplore and Web of Science databases. This review is purely narrative and not meant to be an exhaustive systematic review of these papers. The search term used to identify papers was “stroke rehabilitation” AND (“AI” OR “Artificial intelligence” OR “machine learning”). Papers were then manually screened to ensure that described systems provide some form of interaction with the patient as well as describing the system, machine learning model and results in sufficient detail. This eliminated diagnosis, prognosis and purely movement evaluation based systems as these would not facilitate any form of learning with the patient. Only papers from 2020 onwards were considered to ensure that the review is relevant to state-of-the-art research.

## 5 Results

**Figure 1:**
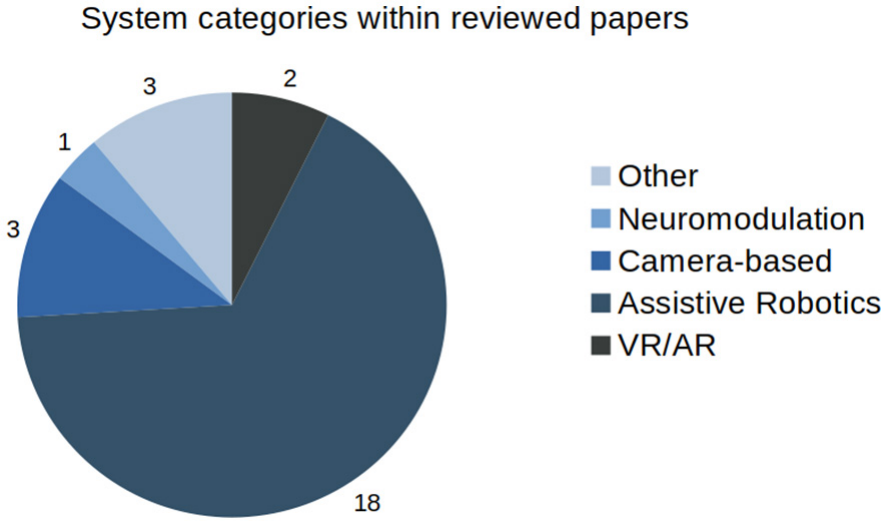
Proportion of each category of rehabilitation technology represented within this review.

**Figure 2:**
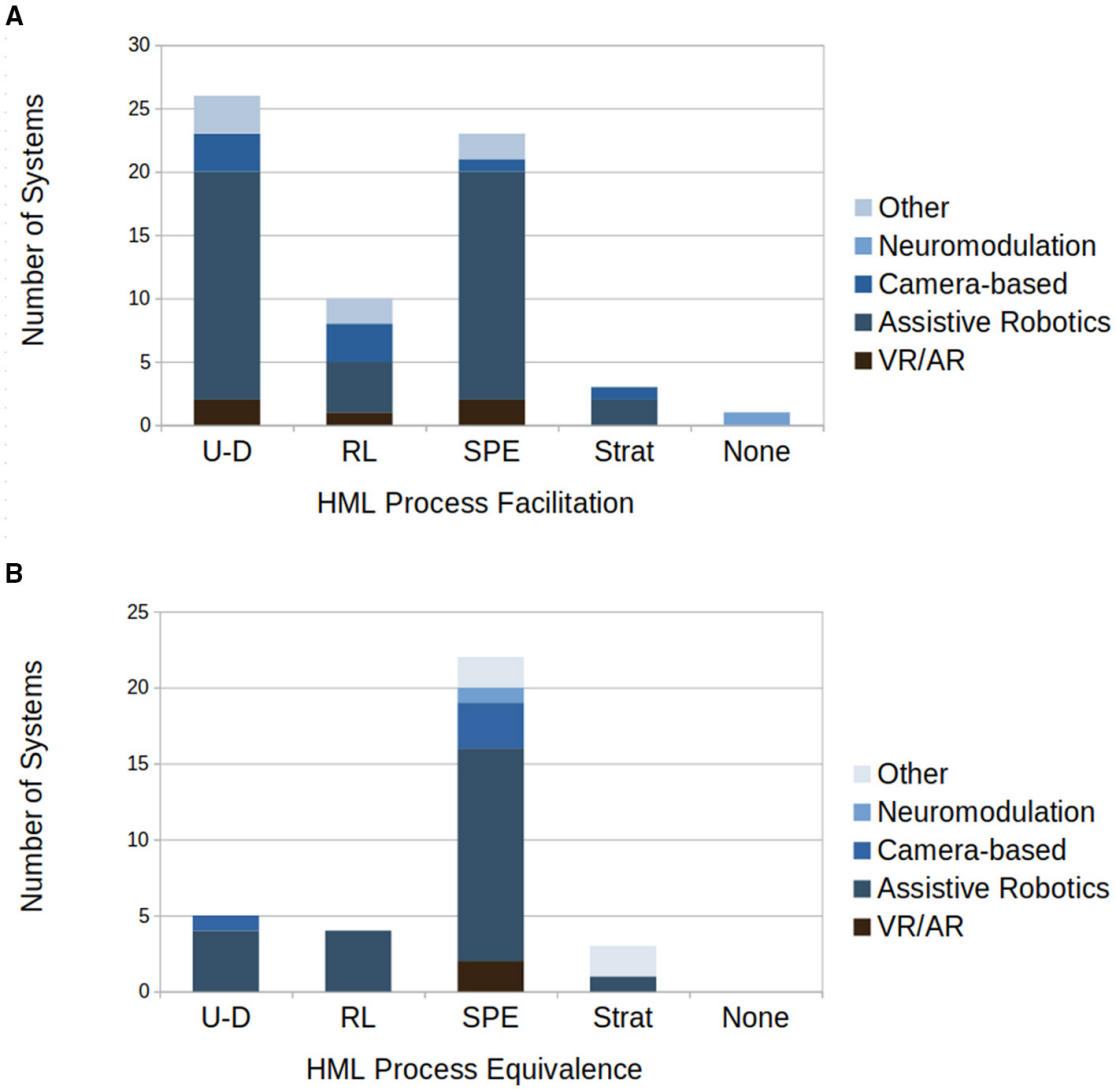
**(A)** Number of systems which facilitate each core HML process within the reviewed cohort. Some systems facilitate multiple HML processes and thus are counted in multiple bars. **(B)** Frequency of models corresponding to each core HML process within the reviewed cohort. Some systems utilize multiple, functionally different, models and thus are counted in multiple bars. (UDL, Use-dependent learning; RL, Reinforcement learning; SPEBL, SPE-based learning; SBL, Strategy-based learning.

In this section, the results of the review will be described. Visual summaries of the type of systems reviewed as well as the HML facilitation and equivalence within these systems can be seen in Figures 1, 2.

### 5.1 Assistive robotics

Assistive robotics (see Table 4) formed the largest group of rehabilitation technologies reviewed, encompassing both end-effector and exoskeleton-based systems. Across this cohort, machine learning models are primarily deployed for movement analysis and prediction, enabling adaptive physical assistance during therapy sessions. These models rely on a variety of sensor modalities, reflecting the ongoing uncertainty around which sensor streams best capture motor intention in stroke populations. Beyond movement prediction, systems also incorporated models for gesture recognition, compensation detection, task difficulty adaptation, and in some cases movement resistance, highlighting a trend toward more personalized and context-sensitive rehabilitation strategies.

**Table 4:** AI-driven assistive robotics systems and their HML mappings.

Name	System	ML used	ML purpose	HML facilitation	HML categorization	Research stage; sample
Castiblanco et al. (76)	EMG-driven Assist-as-needed Exoskeleton for hand rehabilitation	K-NN + ANN	K-NN for hand motion intention classification of EMG signals, ANN for mapping k-NN features to fuzzy logic membership levels	Use-dependent, SPE-based	Use-dependent, SPE-based	Tested on impaired participants; *n* = 24 (20 healthy + 4 stroke)
Guo et al. (70)	An EMG-driven hand rehabilitation glove for mirror therapy via gesture recognition	1D Convolutional neural network (CNN) or InceptionTime	Gesture classification	Use-dependent, SPE-based	SPE-based	Tested on healthy participants; *n* = 8
Jonna et al. (80)	A pressure sensor based 6-DoF end-effector robot for both upper-extremity and lower-extremity rehabilitation exercises	CNN	Intention prediction through pressure sensor	Use-dependent, SPE-based	SPE-based	Tested on healthy participants; *n* = 8
Khan et al. (73)	A 3-DoF pneumatically controlled assist-as-needed platform for ankle rehabilitation exercises	PPO	Energy-informed trajectory generation	Use-dependent, SPE-based	Reinforcement	Tested on impaired participants; *n* = 10 (mild stroke)
Kopke et al. (77)	An EMG-driven rehabilitation robot for AAN planar support during reaching movements	LDA	Window-based classification of movement type and speed	Use-dependent, SPE-based	SPE-based	Tested on impaired participants; *n* = 5 (mod. stroke)
Lee et al. (104)	A seat with varying amounts of stability for trunk rehabilitation	XGB Regressor	Using demographic data and initial trial data to output damping gain values	Use-dependent, SPE-based	Strategy-based	Tested on healthy participants; *n* = 37
Li et al. (78)	A controller for an upper extremity rehabilitation robot capable of AAN rehabilitation as well as resistive control	Particle swarm optimization (PSO)-SVM + LSTM-KF	Classify rehab stage + predict torques from EMG/kinematics	Use-dependent, SPE-based	SPE-based	Tested on healthy participants; *n* = 10
Naznin et al. (72)	A sensor-based glove used in gamified rehabilitation via gesture recognition	SVM/K-NN	Classify healthy vs unhealthy hand movements	Use-dependent, SPE-based, Reinforcement	SPE-based, Use-dependent	Tested on impaired participants; *n* = 68
Rezayat Sorkhabadi et al. (74)	Proposed AAN control algorithm for gait rehabilitation via a 1-DoF knee exoskeleton	GMM	To learn and reproduce therapist interaction torques for adaptive knee assistance	Use-dependent, SPE-based	SPE-based	Proof-of-concept; 4 stroke (data collection only)
Tang et al. (71)	A finger-angle driven AAN pneumatic glove for hand rehabilitation exercises	GP	To predict finger angle dynamics for adaptive pressure control	Use-dependent, SPE-based	SPE-based	Tested on impaired participants; *n* = 6 (3 healthy + 3 stroke)
Xie et al. (75)	A pneumatically controlled, 3-DoF AAN platform for ankle rehabilitation exercises	T-S Fuzzy NARX	Prediction of pneumatic muscle actuator hysteresis behavior for actuator control	Use-dependent, SPE-based	SPE-based	Tested on healthy participants; *n* = 5
Huo et al. (69)	AAN, 4-DoF, lower-body exoskeleton driven by interaction torque for gait rehabilitation	Adaptive NN, GMM Or Expectation maximization (EM)	Adaptive NN for short-term motion estimation, GMM/EM for longer-term joint position prediction	Use-dependent, SPE-based	SPE-based	Tested on healthy participants; *n* = 2
Pezeshki et al. (65)	7-DoF AAN robot arm attached at the hand used in a cooperative two-player game	Policy-iteration RL + Radial basis function (RBF)-NN	RL for assistance policy optimization, RBF-NN for action value approximation	Use-dependent, SPE-based, Reinforcement, Strategy-based	Reinforcement, SPE-based	Tested on healthy participants; *n* = 4
Asl et al. (105)	Interaction-torque, AAN control scheme tested on an upper-limb exoskeleton but applicable to other devices	ANN	Generates intermediary velocities between the desired motion and the actual motion	Use-dependent, SPE-based	SPE-based	Tested on healthy participants; *n* = 3
Luciani et al. (68)	AAN 9-DoF Upper-limb Exoskeleton (ANYexo 2.0) trained on therapist–patient interaction forces	Nearest-neighbor retrieval	Nearest-neighbor retrieval for therapist-force mimicry	Use-dependent, SPE-based	Use-dependent	Tested on healthy participants; 1 patient-therapist pair
Pareek et al. (66)	AAN rehabilitation robot with a pen-shaped handle for wrist and hand rehabilitation	SAC	SAC for assistance-gain modulation	Use-dependent, SPE-based, Reinforcement	Reinforcement	Tested on healthy participants; *n* = 8
Hou et al. (79)	3-DoF Wrist rehabilitation robot with screen displaying reference and actual trajectory	GMM/GMR, Kernelised movement primitives (KMP), REPS	GMM/GMR for generating human-preferred trajectories, KMP for deforming trajectories via via-point constraints, REPS for context-dependent policy optimization	Use-dependent, SPE-based, Strategy-based	Reinforcement, SPE-based, Use-dependent	Tested on healthy participants; *n* = 11
Martínez-Pascual et al. (67)	An AAN robot with a handle end effector for table-top, upper extremity, gamified rehabilitation	1D-CNN with attention mechanism	Classify movements into “needing assistance” or not based on therapist data	Use-dependent, SPE-based, Reinforcement	SPE-based	Tested on impaired participants; *n* = 8 (mixed neurological)

Assistive robotic systems reviewed in this category target a range of motor impairments across both the upper and lower extremities. Several systems focused on upper-limb rehabilitation, including robotic arms, end-effector devices, and wearable exoskeleton, are designed to support reaching, wrist manipulation, or hand function. End-effector robots typically guide the patient's limb through handles or attachment points during goal-directed tasks such as reaching or trajectory tracking (65–67). In contrast, exoskeleton-based systems provide joint-level assistance through articulated mechanical structures aligned with the user's anatomy, allowing direct modulation of joint torques during rehabilitation exercises (68, 69). Several systems also target hand and finger rehabilitation, using pneumatic gloves to support finger flexion and extension during repetitive grasping tasks or sensorized gloves to enable mirror therapy via assistance based on hand gestures from the non-paretic hand (70–72). Lower-limb rehabilitation was represented by robotic platforms designed for ankle training or lower-body exoskeletons designed for gait-related assistance, all of which support repetitive joint movements during rehabilitation exercises via AAN control (73–75). AAN control is prevalent throughout these systems, where the robot provides minimal support during successful movement execution and increases assistance when deviations from the desired motion occur.

To enable adaptive assistance, these systems relied on a variety of sensing modalities to monitor user intention and movement performance. Electromyography (EMG) was frequently used to estimate muscle activation patterns and infer intended movement, allowing the robot to trigger or modulate assistance based on the user's voluntary effort (70, 76–78). Other systems utilized interaction forces, where forces exchanged between the patient and the robotic device were measured to estimate user contribution and regulate assistive torques. This occurred through both pressure plate forces as well as joint torques in multiple DoF systems (68, 69, 74). In addition, several platforms relied on kinematic or positional sensing, including joint encoders, and trajectory tracking systems to monitor limb motion and assess task performance (67, 71). In some of the reviewed systems, sensing modalities were combined to provide machine learning models with multimodal input data for movement classification, intention prediction, and adaptive assistance control (78).

Together, the AAN methods and different sensory modalities allow for more active participation and facilitation of HML mechanisms. These systems mainly facilitate use-dependent learning, since their central function is to assist in large volumes of repetitive movement training. In addition, SPE-based learning is often promoted by their assistive and corrective nature, which allows patients to experience normative kinematic patterns and thereby strengthen internal forward models. A small collective of papers also introduce elements of reinforcement learning, where tactile and haptic feedback acts as a reward-like signal contingent on movement performance (66, 67). Knowledge of results is also provided through gamified rehabilitation and visualization of task performance within some papers (65, 66, 79).

In terms of the machine learning approaches used, most models used align with the definitions of SPE-based or strategy-based learning with use-dependent learning appearing only in systems that employed unsupervised clustering algorithms such as K-NN. Predictive models such as GMMs, GPs, T-S fuzzy NARX, LSTM–Kalman filters (LSTM-KF), adaptive neural networks, and deep time-series CNNs predominantly reflected SPE-based learning, owing to their emphasis on probabilistic prediction and minimization of prediction error. Typical use cases included motion/intention prediction. While SVM and LDA do use explicit decision boundaries, the functionality of their training more aligns with SPE-based learning, thus only a single system, using an XGB Regressor, could be characterized as similar to strategy-based learning. Three systems utilized models with characteristics equivalent to use-dependent learning, through the use of K-NN models. Reinforcement learning frameworks were less common, with only a small subset employing PPO, SAC, policy iteration or REPS for adaptive assistance or task-level policy optimization (65, 66, 73, 79). Systems that utilize multiple models, with different HML representations, were rare with only a few having two HML processes represented and only one having 3 HML processes represented. For consistency, this review focuses on the final or best-performing models reported in each study.

In terms of machine learning results, the majority of systems demonstrated strong classification or prediction performance. For example, CNN based systems demonstrated, CNN-based movement classification reported accuracies above 98% (80), and deep learning models such as InceptionTime reached around 91% for gesture recognition (70) and CNNs using attention mechanisms demonstrated accuracies of 91% and F1 scores of 75% (67). Other approaches, including K-NN and fuzzy models, achieved accuracies between 85% and 95% depending on the task (76). Regression-based methods such as Gaussian Process models and LSTM–KF provided low error rates in torque estimation and trajectory prediction (71, 78). Additionally, task-level contextual policy search increased success rates from approximately 30% to 96% after 150 episodes with REPS (to about 60% with GP-UCB) (79), an SAC-based assist-as-needed controller produced significant T1 → T3 tracking-error reductions in 3/4 participants while 0/4 improved under a rule-based baseline (66), and a memory-based nearest-neighbor torque retrieval model reduced therapist-force isolation error by up to 92% with a maximum estimation error of 0.31 Nm at the shoulder joint (68). Collectively, these results indicate that within the reviewed systems, in controlled experimental settings, machine learning algorithms are capable of reliably interpreting sensor data and adapting robotic assistance to user performance.

Patient validation was more limited and typically involved small cohorts. Studies reported decreases in shoulder muscle activity during assisted reaching (77), and increases in range of motion with robotic support (77). Other findings included improvements in hand coordination and reductions in muscle co-contraction indices following multi-session glove training (71). Only one study reported validated clinical outcome measures, with improvements observed in the Fugl-Meyer Assessment and Action Research Arm Test after repeated training sessions with a soft pneumatic glove (71). While these results highlight promising early evidence of functional benefit, the majority of the reviewed studies remain at the feasibility stage, reporting biomechanical or behavioral improvements rather than long-term clinical recovery.

### 5.2 VR/AR systems

With assistive robotics consisting of over half the reviewed papers, VR and AR (see Table 5) form a subset of the remaining rehabilitation technologies, designed to improve accessibility, engagement, and immersion in motor training. Unlike passive exercise platforms, these systems situate repetitive practice within interactive or visually augmented environments, potentially enhancing motivation and sensorimotor feedback quality. Applications included augmented reality smart glasses for mirror therapy (81) and a depth camera–based VR platform for posture monitoring during exercise (82).

**Table 5:** AI-driven VR/AR systems and their HML mappings.

Name	System	Machine learning used	Purpose	HML facilitation	HML categorization	Research stage; sample
Chang et al. (81)	A2Mirror AR smart glasses for visualizing hand movement during mirror therapy	MediaPipe CNN	Hand gesture recognition	SPE-based, Use-dependent	SPE-based	Tested on impaired participants; *n* = 3 (stroke)
Maskeliun̄as et al. (82)	BioMacVR for posture/motion analysis and provision of feedback	Random forest	Exercise classification	Use-dependent, Reinforcement	SPE-based	Proof-of-concept; *n* = 0

These systems predominantly facilitate use-dependent learning, supporting high volumes of repetitive training in engaging settings. SPE-based learning is facilitated where mirrored visual feedback helps recalibrate sensory predictions, as seen in Chang et al. (81). Elements consistent with reinforcement learning appear when game tasks or posture corrections provide contingent feedback, such as success/failure indicators or classification of correct versus incorrect movements (82).

In terms of HML similarity, the models align solely with SPE-based learning, reflecting the fact that machine learning in both systems is applied to movement classification, where model updating is driven by prediction error minimization rather than explicit rule construction or trial-and-error policy search.

Both systems demonstrated strong technical performance. The A2Mirror system applied MediaPipe CNNs for finger flexion–extension and thumb-to-finger gesture recognition to support AR-based mirror therapy (81). The BioMacVR platform combined CNN pose classifiers with random forest classifiers for posture analysis, reporting a 23 ms average response time and real-time discrimination between correct and incorrect postures during exercise (82).

Clinical results remain limited. A2Mirror was tested with three post-stroke patients over a three-month intervention, with significant improvements reported in FMA-UE subscale scores for finger flexion–extension and thumb-to-finger tasks on the affected side (81). BioMacVR validated its technical classification ability in healthy participants but did not report clinical outcomes in stroke patients (82). Overall, while the reviewed ML-driven VR/AR systems demonstrate technical feasibility and some preliminary evidence of functional benefit, broader clinical validation remains at the proof-of-concept stage.

### 5.3 Vision-based systems

Computer vision and AI-based feedback systems (see Table 6) constituted another group of rehabilitation technologies reviewed, using cameras and pose-estimation pipelines to monitor movement and provide corrective guidance without wearable sensors. Unlike the sensor-dependent systems reviewed elsewhere in this paper, these approaches offer a less encumbered interaction, relying instead on visual capture of body landmarks or facial features to assess movement quality. Applications included OpenPose-based posture detection for rehabilitation exercises (83), a hybrid fuzzy-logic and K-NN model for tracking lower-limb joint angles during exercise (84), and an AI-driven video-game platform using facial landmark tracking for dysphagia therapy (85).

**Table 6:** AI-driven camera-based systems and their HML mappings.

Name	System	Machine learning used	Purpose	HML facilitation	HML categorization	Research stage; sample
Chen and Yang (83)	Posture quality analysis for feedback during remote rehabilitation	OpenPose CNN	Pose ID + feedback	Use-dependent, Reinforcement	SPE-based	Tested on healthy participants; unspecified
Das et al. (84)	Rehabilitation exercise detection and counting for visualization of results and feedback	MediaPipe CNN + Fuzzy logic, KNN	KNN for exercise labeling, MediaPipe CNN + Fuzzy logic for categorizing exercise correctness and performance	Use-dependent, Reinforcement	Use-dependent, SPE-based	Tested on healthy participants; *n* = 30
Zhang et al. (85)	Facial muscle detection for control of a gamified rehabilitation system	MediaPipe CNN	Detection of specific facial landmark positions	Use-dependent, Reinforcement, Strategy-based	SPE-based	Clinical RCT; *n* = 32 (stroke)

From a HML perspective, these systems predominantly facilitate use-dependent learning. As with most rehabilitation technologies, they encourage large volumes of repetitive, task-specific practice. SPE-based learning is supported where visual overlays or game feedback expose discrepancies between intended and executed movement, helping recalibrate internal predictions (83, 85). Reinforcement-like processes arise through feedback such as video game scores or corrective instructions, and strategy-based learning is facilitated within (85) in tasks that require patients to adapt swallowing approaches across exercises (84, 85).

In terms of HML similarity, the machine learning models employed largely align with SPE-based learning with only one paper incorporating a use-dependent learning like method. Posture recognition and skeleton tracking using CNNs reflect SPE-based learning, updating weights through label/classification prediction error (83–85). K-NN classifiers reflect the repetition-driven bias of use-dependent learning. The usage of fuzzy-logic within exercise classification leans toward a strategy-based learning similarity, however, the rules themselves are not updated and thus it does not qualify (84).

Das et al. trained a K-NN model on datasets curated by rehabilitation specialists, achieving 97% accuracy for hip flexion, 92% for hip external rotation, and 91% for knee extension, while their hybrid fuzzy-logic model supported joint-angle tracking with a lowest measurement error of 0.34° and very high reliability (84). Zhang et al. and Chen and Yang did not describe their machine learning model results and instead focused on user outcomes instead.

For clinical results, Chen and Yang demonstrated significant improvements in posture execution before and after system usage. However, they did not report validated clinical scales or specify if the participants were healthy controls or stroke patients (83). By contrast, Zhang et al. produced significantly greater improvements in the Gugging Swallowing Screen (GUSS) at both post-intervention and one-month follow-up. Furthermore, significant gains were observed in Functional Oral Intake Scale (FOIS), Mini-nutritional Assessment Short Form (MNA-SF), and the Swallowing Quality of Life Questionnaire (SWAL-QOL), with no between-group difference in Standard Swallowing Assessment (SSA). This is accompanied by higher patient adherence, acceptance, and satisfaction (85). Overall while clinical trials are still limited, Zhang et al. provide promising evidence for the use case of AI-driven rehabilitation games for recovery.

### 5.4 Neuromodulation

A single Neuromodulation paper incorporating machine learning was found through the reviews search criteria (see Table 7). Ye et al. (86) introduced a stroke rehabilitation framework that combines non-invasive brain stimulation (NIBS) with machine learning to personalize stimulation protocols. The system employed recurrent neural networks (RNNs), including LSTM variants, to analyze sequential Electroencephalogram (EEG) and functional magnetic resonance imaging (fMRI) data and predict recovery trajectories in real time.

**Table 7:** AI-driven neuromodulation systems and their HML mappings.

Name	System	Machine learning used	Purpose	HML facilitation	HML categorization	Research stage; sample
Ye et al. (86)	A Non-invasive brain stimulation system using ML on fMRI/EEG signals for closed-loop stimulation protocols	RNN	Decode neural time-series to drive adaptive stimulation	No direct facilitation, increases plasticity and/or excitability	SPE-based	Proof-of-concept; *n* = 0

From a HML perspective, this approach does not directly facilitate any HML processes but instead encourages neural plasticity and excitability. When comparing the model used with the HML processes, the RNN model used is most analogous to SPE-based learning. This is due to stimulation parameters are adjusted by comparing expected neural activity against measured signals.

In terms of machine learning results, the framework reported strong performance in a simulation environment integrating prior clinical datasets. Prediction of recovery trajectories achieved 97.65% accuracy, functional recovery score prediction reached 98.62%, and overall rehabilitation success and clinical efficiency were both reported at 93.21%. These scores represent performance indicators based on secondary data and simulation, rather than direct outcomes from a new clinical trial.

### 5.5 Other systems

A smaller group of studies explored approaches that do not sit neatly within robotics, VR/AR, Neuromodulation, or camera-only categories (see Table 8). These include an AI therapist that reproduces expert verbal cues during robot-assisted gait training (87), a glove-based self-training motivator that senses hand exercises and reminds users to practice (88), and a socially assistive robot that personalizes corrective feedback for upper-limb exercises (89).

**Table 8:** Other AI-driven rehabilitation systems and their HML mappings.

Name	System	Machine learning used	Purpose	HML facilitation	HML categorization	Research stage; sample
Chang et al. (87)	Verbal cueing (gait)	Neuro-fuzzy	Cue selection (6 verbal options)	Use-dependent, Reinforcement, SPE-based	Strategy-based	Tested on impaired participants; *n* = 58 (stroke)
Lee et al. (89)	Robot exercise coaching	NN + rule-based ensemble model	Categorize ROM, movement smoothness and compensation to provide feedback	Use-dependent, Reinforcement	Strategy-based, SPE-based	Tested on impaired participants; *n* = 15 (stroke)
Zubrycki et al. (88)	Hand rehab glove (IMU + tablet)	CNN	Exercise detection	SPE-based, Use-dependent	SPE-based	Tested on healthy participants; *n* = 4

These systems facilitate multiple HML processes. The AI therapist encourages use-dependent learning by driving high-volume gait practice and reinforcement learning via timely, task-dependent verbal cues (87). The glove system promotes use-dependent learning through repeated home exercises and provides feedback that supports SPE-based learning as users compare intended and detected movements to reduce error. The socially assistive robot supports use-dependent repetition, SPE-based recalibration via corrective prompts, and strategy-based learning by asking patients to adjust compensatory patterns during tasks (89).

In terms of HML correspondence, the core ML models align primarily with SPE-based and strategy-based learning. The AI Therapist's neuro-fuzzy controller is most similar to strategy-based learning, since it utilizes explicit rules for feedback provision and these are updated through learning (87). The glove's CNN-based exercise detection aligns most strongly with SPE-based learning through its use of prediction and prediction error in training. The robot exercise coach utilizes both an ANN and a rule-based model for movement analysis from the former to drive performance categorization, which in turn determines the feedback provided by the latter. Functionally these are most analogous to SPE-based learning due to learning through prediction error and strategy-based learning due to the use of explicit rule-based feedback provision (88, 90).

The results of the machine learning models were that the AI Therapist reproduced six categories of therapist verbal cues with 93.7% accuracy over 1,812 test samples derived from 693 gait-training sessions with 58 stroke patients (87). The glove system achieved 91.3% accuracy and F1 = 91.6% for known users, with performance reducing to about 78% accuracy and F1 ≈ 81% for new users, highlighting generalization challenges (88). The socially assistive robot improved motion-assessment performance, with F1-scores rising from 0.7447 to 0.8235 after personalization using each patient's unaffected-side data, and frame-level compensation detection improved via ensemble voting (*p* < 0.01) (89).

While some systems were tested on patients, none of the studies conducted clinical validation using recognized clinical scales.

## 6 Discussion

### 6.1 Synthesis of key findings

Across modalities, most of the reviewed systems in stroke rehabilitation facilitate use-dependent learning by enabling repetitive movement training, with engagement of SPE-based learning and reinforcement learning and strategy-based learning being secondary. Within the review, explicit, multi-process modeling of HML within a single controller remains uncommon. Assistive robotics constitutes the largest share of studies in the review, whereas VR/AR, camera-only, neuromodulation, and socially assistive/other systems are represented by smaller cohorts and mainly via proof-of-concept evaluations. Despite strong technical metrics, many of the papers remain as demonstrations of feasibility, with the cohort showing a minimal amount of clinical results.

### 6.2 Evidence strength and gaps

The majority of the reviewed studies report accurate movement analysis/prediction and biomechanical improvements (e.g., compensation reduction, muscle activity changes), but patient cohorts are small and validated clinical measures of functional outcomes are infrequently reported; only one glove-based study reported improvements on FMA-UE and the Action Research Arm Test (ARAT). VR/AR systems demonstrate high technical performance and preliminary functional benefit (e.g., A2Mirror with three post-stroke patients), yet broader clinical validation remains at the proof-of-concept stage. Across modalities, research into the retention of performance gains after engagement with the rehabilitation systems remains limited.

Due to limited clinical results, it becomes difficult to conclude from these papers that the use of ML within rehabilitation robotics results in improved long-term motor performance gains over non-ML systems. However, some indirect links can be made through prior research. Evidence indirectly shows that active participation within motor practice results in higher retention than passive practice through greater and more distributed cortical reorganization (91). This information can be used to make the association that assistive robotics that use ML-based assist-as-needed controllers may result in higher retention compared to fixed-assistance, guiding, robotics.

A further argument for the case of ML-driven rehabilitation systems is the provision of adaptive feedback. Feedback in the form of both knowledge of performance (KP) and knowledge of results (KR) is demonstrated within the reviewed papers. Game-based systems such as within Zhang et al. provide a great example of symbolic feedback, providing KR in the form of video-game scores (85). KP is clearly provided by the A2Mirror glasses in Chang et al. as well as the other camera-based systems, informing participants on their motor performance when completing rehabilitation tasks (81–84). Facilitating reinforcement learning mechanisms, this provides a fruitful avenue for future research in the form of smart feedback based on vision or other sensor-based systems. Together, these observations suggest the use of “smart” KP+KR, delivered on adaptive (not constant) schedules as too much feedback can have a negative impact on motor performance and learning (92). Inclusion of such mechanisms into assistive robotics may allow for more “complete” facilitation of the HML processes.

### 6.3 HML functional similarity

One of the core contributions of this narrative review is the analysis of the presence of functional representations of HML processes within the reviewed systems. This is with the hypothesis that designing systems that both learn *the way* therapists learn as well as learning *what* they learn, will be more robust and have better functional outcomes. Due to the heterogeneous measures of performance within the models, limited clinical validation, and narrative nature of this review, the results of this review can not be clearly stated to support this hypothesis. Thus, we present this perspective as a testable design hypothesis rather than a confirmed effect, and we outline a research agenda to determine whether a multi-process, therapist-inspired controller can deliver superior robustness and functional benefit.

Based on the background research we make the assumption that therapists routinely integrate the four HML processes in a context-dependent way. They use SPE-based corrections for unexpected patient responses, reinforcement signals from success, effort, or discomfort to adjust assistance, strategy-based rules to set safe, goal-directed starting policies for new patients, and use-dependent consolidation to streamline patterns that work. This seamless arbitration—switching and weighting processes as task demands, uncertainty, and patient state change, may help explain the gap between therapist adaptability and current assistive robotics, which often only model one process as a part of the system and often not with the intent of directly replicating therapist behavior.

Therefore, we propose that being able to computationally represent all four processes within an adaptive system may theoretically bridge the gap between therapist adaptability and rehabilitation technology adaptability.

### 6.4 Closed-loop neuromodulation for stroke

Among the technologies represented in this review, AI-driven neuromodulation was absent, despite its growing presence in the broader stroke rehabilitation literature (93). This is notable given prior evidence of the effectiveness of neuromodulation for functional recovery in stroke, as well as emerging evidence that closed-loop neuromodulation may outperform open-loop approaches in terms of dosage efficiency in other neurological conditions.

Dawson et al. provide a comprehensive review of neurostimulation in stroke rehabilitation, detailing methods trialed in clinical studies. Vagus nerve stimulation alongside high-intensity rehabilitation training was found to increase FMA upper extremity scores significantly more than rehabilitation training alone. The review also detailed the effectiveness of pharyngeal electrical stimulation for treating dysphagia, showing that patients were significantly less likely to require a tracheotomy tube after treatment compared to sham participants (93).

Evidence for closed-loop neuromodulation has been demonstrated in other neurological conditions. Little et al. found that an adaptive deep brain stimulation system using BCI feedback produced an over 50% increase in motor performance in Parkinson's patients while reducing stimulation time by 56%, compared to continuous deep brain stimulation (94). While this evidence comes from a non-stroke population, it motivates consideration of similar approaches in stroke rehabilitation contexts.

In many current neuromodulation applications, dosage and timing schedules are pre-defined by clinicians rather than dynamically adjusted based on real-time physiological feedback (95). This open-loop approach risks non-specific or excessive stimulation, which may contribute to side effects such as mania or hypomania, particularly when stimulating deeper brain structures with less clearly defined functional boundaries, such as the basal ganglia (96). A closed-loop system integrated with BCI feedback could in principle improve both safety and efficacy by tailoring stimulation parameters to the user's real-time neural, physiological, and emotional state, though clinical validation of such approaches in stroke remains limited. In this context, the HML framework offers a useful lens for conceptualizing how machine learning might be integrated into neuromodulation. Reinforcement learning models could analyze motor performance during tasks and use this as feedback to reward stimulation parameters associated with the greatest functional gains. Strategy-based learning could similarly be used to formulate generalized baseline stimulation strategies tailored to the level and type of impairment, which could then be refined over the course of treatment.

### 6.5 Benchmarking, personalization, and generalization

A core challenge in the evaluation of AI-driven rehabilitation systems is the lack of standardized benchmarking practices. The reviewed studies often rely on internally defined technical metrics, making cross-comparison between systems difficult. To ensure clinical relevance, benchmarking should incorporate established clinical outcome measures, such as the Fugl-Meyer Assessment (FMA), alongside long-term retention studies that capture durability of functional gains (97).

Furthermore, benchmarking must extend beyond immediate efficacy to capture adaptability over time. Longitudinal trials are required to evaluate how systems respond to fluctuations in patient performance, such as fatigue or recovery progression. Examples include adaptive assistance-as-needed (AAN) robotics and gamified rehabilitation protocols, which dynamically adjust task difficulty based on patient response.

Generalization remains another under-explored aspect of performance within this review. While some systems report promising transferability across participants, these findings are typically based on small and homogeneous cohorts, often restricted to similar impairment levels. To achieve true generalizability, future studies should include diverse patient populations spanning a wide range of impairments, recovery phases, and demographic characteristics. This would enable more robust conclusions regarding system scalability and clinical applicability.

In turn future research should aim to standardize the following benchmarks: (i) standardized tasks/protocols, (ii) common clinical scales, (iii) retention probes, and (iv) cross-patient generalization tests spanning impairment diversity. This should enable better comparison in the effectiveness of each system.

### 6.6 Limitations of this review

This review was conducted as a narrative synthesis rather than a systematic review, which introduces selection bias and limits generalizability. Database scope, inclusion choices, and language constraints may have omitted relevant studies. Heterogeneity in tasks, cohorts, sensors, and outcome measures, combined with the predominance of feasibility studies, precluded formal meta-analysis.

The proposed mapping of ML models to HML equivalence and the identification of facilitation mechanisms, while grounded in operational definitions, remain partly interpretive and may be sensitive to reporting quality and task context. This limitation is further compounded by the discontinuous nature of machine learning relative to HML. Many models classified here as SPE-equivalent are trained offline on labeled datasets and deployed as fixed inference systems during therapy, meaning no weight updates occur during the rehabilitation interaction itself. HML, by contrast, is continuous and does not have distinct phases of learning and inference/deployment. While therapists also undergo formal training prior to working with patients, this does not confine their learning to that period—they continue to adapt and refine their assistance strategies across every patient interaction (7). A fixed, deployed model has no analogous capacity for ongoing adaptation, meaning the SPE-based equivalence assigned to such models reflects their training mechanism rather than their behavior during deployment. This is in contrast to sequential models such as LSTMs, which retain a form of short-term adaptation through their recurrent state even after deployment. The current mapping does not distinguish between these two types of SPE-like learning, representing a limitation that future refinements should address.

Moreover, several domains (e.g., closed-loop neuromodulation and large-scale VR/AR clinical trials) are under-represented in the reviewed literature, which constrains the strength of modality-specific conclusions. Finally, many included studies emphasize algorithmic performance over validated functional outcomes and long-term performance retention, limiting inference about real-world clinical effectiveness.

## 7 Conclusion

Across the described assistive robotics, VR/AR, camera-based feedback, and emerging neuromodulation approaches, the reviewed, AI-driven rehabilitation systems appear to predominantly facilitate use-dependent learning within patients, with more limited but growing evidence for the facilitation of SPE-based, reinforcement-driven, and strategy-based learning. However, explicit implementation of multi-process HML facilitation within a single control framework remains uncommon, and many of the reviewed studies continue to emphasize technical metrics rather than standardized clinical outcomes or retention assessments.

Further investigation into promising fields such as closed-loop neuromodulation, adaptive feedback modalities (e.g., knowledge of performance and knowledge of results) and gamification of rehabilitation could serve as avenues for future research. Furthermore, a distinct and under-explored direction in the reviewed papers lies in the modeling of therapist HML. We propose the hypothesis that therapist-inspired, multi-process control architectures, capable of representing and arbitrating between sensory prediction error corrections, reinforcement-based adaptation, strategy formulation, and use-dependent bias, may provide greater robustness and functional benefit than single-process approaches. Progress toward this goal and measuring its outcomes will require: (i) pre-registered, adequately powered trials incorporating validated outcomes with multi-time-point follow-ups; (ii) transparent technical reporting with external validation and safety evaluation; (iii) the establishment of shared benchmarking protocols that integrate clinical scales with retention and generalization assessments. Collectively, these steps would likely help to advance the field from feasibility-focused demonstrations toward reproducible, patient-relevant outcomes that meet the standards required for clinical translation.
